# Angiosarcoma of the Liver: A Radiological Alarm for Radiologists and Hepato-Pancreato-Biliary (HPB) Surgeons

**DOI:** 10.7759/cureus.87622

**Published:** 2025-07-09

**Authors:** Evangelia Florou, Archana Abarnadevikarthikeyan, Stephen M Gregory, Parthi Srinivasan, Andreas Prachalias

**Affiliations:** 1 Hepato-Pancreato-Biliary Surgery, King's College Hospital, London, GBR; 2 Hepato-Pancreato-Biliary Interventional Radiology, King's College Hospital, London, GBR; 3 Hepato-Pancreato-Biliary Surgery and Liver Transplantation, London Bridge Hospital, London, GBR

**Keywords:** bening liver tumors, biliary cystadenoma, cavernous haemangioma, hepatic angiosarcoma, liver angiosarcoma, liver cyst, spontaneous rupture

## Abstract

Hepatic angiosarcoma (HA) is a rare and aggressive malignancy. Radiological findings are non-specific and often mimic benign liver pathologies. Patients’ rapid clinical deterioration is often alarming, leading clinicians to a late, futile diagnosis. We present two cases of HA in which presenting symptoms, but more importantly, radiological appearances, were misleading, mimicking liver cysts and cavernous haemangiomas, respectively. Rapid clinical deterioration and speedy radiological evolution of disease spread led to a diagnosis of HA with a dismal prognosis.

A 77-year-old male was diagnosed with an incidental finding of a cystic lesion in the upper abdomen. On contrast-enhanced computed tomography (CECT), the lesion mimicked a complex liver cyst with atypical radiological features, prompting clinicians to treat it with surgical resection for an otherwise unclear malignant pathology. On repeat CECT prior to surgery, the disease explosion with extensive peritoneal spread was surprising. A 79-year-old female presented with right flank pain, and CECT showed features of a cavernous haemangioma on the right liver lobe. The MRI confirmed atypical features of an otherwise benign entity, not long before the patient presented with spontaneous rupture, treated with embolisation. On repeat CECT, findings of new multiple liver lesions representing disease spread led to a biopsy confirming HA.

Radiological appearances of HA are non-specific and may mimic benign liver pathologies, misleading clinicians. Early radiological detection and clinician awareness may lead to timely diagnosis, as complete resection of this aggressive malignancy offers better outcomes. Recognised treatment options appear limited in most cases, and future molecular analyses of this aggressive cancer may help advance systemic therapies.

## Introduction

Hepatic angiosarcoma (HA) is a rare primary liver malignancy, which accounts for only 1-2% of all primary liver malignancies [1-3] and 2% of all sarcomas [1,3]. It affects patients in the sixth decade of life [3,4], and some risk factors, such as former exposure to Thorotrast [5,6], vinyl chloride [7,8], and arsenic compounds [9], have been identified in 25-40%, but in the majority of the cases, no risk factor is identified [3].

HA is not easy to diagnose, as patients are often asymptomatic [1,3]. Symptomatic presentation occurs in 9% of the cases, with non-specific symptoms such as abdominal pain and discomfort, which may be late signs of metastatic disease [3]. Presentations with fulminant acute liver failure or intra-abdominal bleeding due to spontaneous tumour rupture have also been described, with the former being scantly documented in case reports [4,10] and the latter reported in 17-27% of the cases [3,11]. 

Histological confirmation may be challenging [1], as the tumour may be mimicking poorly differentiated carcinoma or undifferentiated sarcoma [1]. 

Surgical resection with negative margins is the only treatment with curative potential [3]. However, early diagnosis is quite rare, and the majority of the cases present with metastatic disease [3]. Systemic treatments do not offer improved prognosis, which remains dismal with a median overall survival of six months [3]. 

Radiologically, HA presents a diagnostic dilemma. Imaging features are often non-specific and may mimic benign hepatic lesions such as liver cysts or cavernous haemangiomas, leading to misinterpretation and delayed diagnosis. Furthermore, the rapid progression between imaging studies may be easily underestimated, masking the underlying malignant nature of the disease [2].

This article examines two cases of HA, underscoring the radiological diagnostic challenges that can arise in the absence of symptoms. In such cases, imaging techniques, typically considered as invaluable tools for early cancer detection, prove ineffective in diagnosing this aggressive and rare form of cancer.

## Case presentation

Case 1 

**Figure 1:**
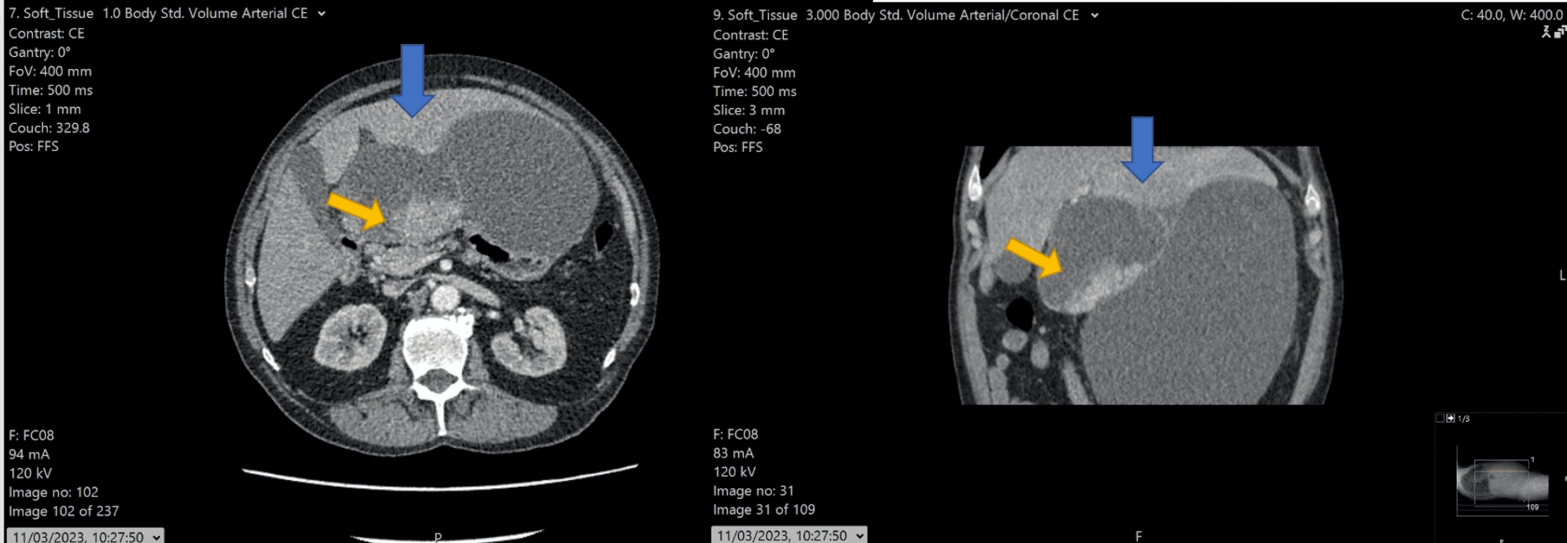
Hepatic angiosarcoma (HA) mimicking a complex liver cyst Axial and coronal imaging of CECT showing a large cystic lesion arising from the left lateral segment of the liver expanding between the liver, pancreas, and stomach (blue arrow). Some soft tissue components are evident (yellow arrow); however, in the context of the bi-lobar cystic lesion, this was interpreted initially as cyst wall complexity. HA: Hepatic angiosarcoma: CECT: Contrast-enhanced computed tomography

A 77-year-old male with a history of hypertension, bronchiectasis, and mild asthma was investigated for suspected lung cancer due to a persistent cough. During the diagnostic workup, which included contrast-enhanced computed tomography (CECT) of the chest, an incidental cystic lesion in the upper abdomen was revealed, a finding that prompted further imaging studies and referral to our tertiary centre. An abdominal CECT revealed a cystic lesion measuring 22 mm in maximum diameter located between the liver and the lesser curvature of the stomach (Figure 1). 

**Figure 2:**
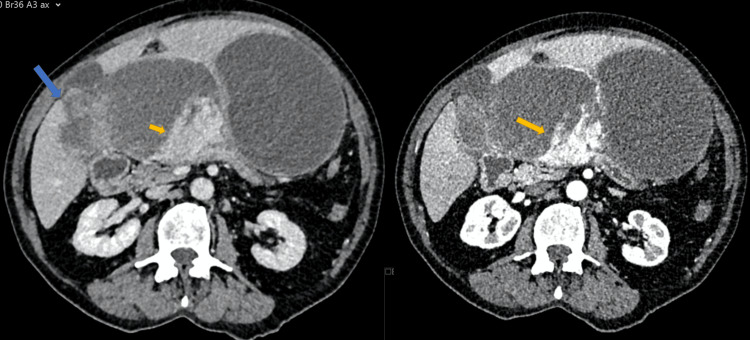
HA mimicking complex liver cyst Axial and coronal imaging of CECT showing the cystic lesion increased in size. On the right, a new solid tissue component infiltrating the liver (blue arrow) is evident along with a further increase in the inter-cystic soft tissue part of the lesion (yellow arrow). On the left, the inter-cystic soft tissue component is enhanced on arterial phase imaging (yellow arrow). HA: hepatic angiosarcoma; CECT: contrast enhanced computed tomography

The case was brought for discussion in the multidisciplinary meeting, as radiological appearances were atypical; thus, diagnosis and management required discussion amongst specialists and consensus. Radiologically, the cyst appeared multilocular with a few internal septations. The differential diagnosis included complicated liver cyst, cystic differentiation of haemangioma, and biliary cystadenoma. Given the atypical features, a consensus was made to proceed to surgical resection in the form of peri-cystectomy for full histopathological evaluation and establishment of a definitive diagnosis. The patient was scheduled for surgery four weeks later. On the preoperative repeat CECT scan, findings were surprising. The cystic lesion appeared doubled in size and had clear features of malignant pathology (Figure 2).

On laparotomy, the CECT findings were confirmed, and multiple sites of peritoneal disease were identified. Core biopsies from the partly solid part of the lesion infiltrating the liver were taken for diagnosis confirmation. The histopathological examination revealed a pleomorphic malignant neoplasm consistent with HA. The patient was referred to the special Sarcoma Oncology centre for further treatment, but succumbed to the disease within four months.

Case 2

**Figure 3:**
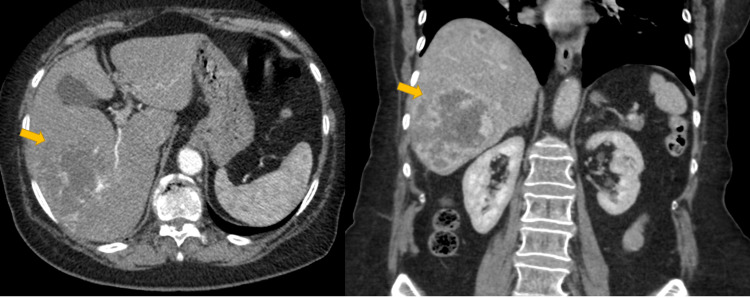
HA mimicking cavernous haemangioma Axial and coronal imaging of CECT showing the lesion with appearances in keeping with cavernous haemangioma (yellow arrow). Interpretation of the lesion as being a cavernous haemangioma was misleading, as the lesion was representing an HA. HA: hepatic angiosarcoma; CECT: contrast-enhanced computed tomography

A 79-year-old female with a history of right-sided breast cancer treated with surgical excision presented with right-sided flank pain. A CECT scan of the thorax, abdomen, and pelvis was performed, which revealed a large lesion in segments V/VI of the liver (Figure 3). The lesion measured 8 cm in maximum diameter and appeared to have internal as well as peripheral nodular discontinuous enhancement with progressive centripetal fill-in. The overall radiological impression was that the lesion represents a somewhat atypical cavernous haemangioma.

**Figure 4:**
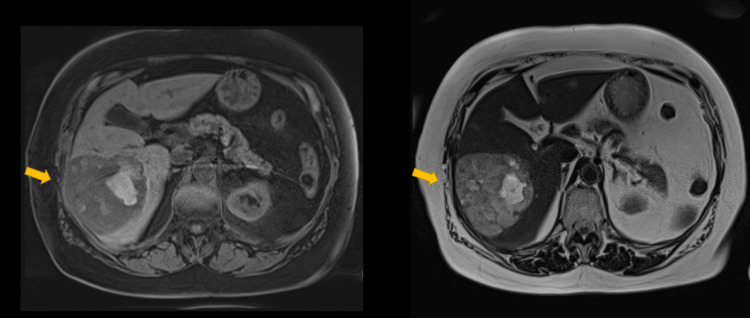
HA mimicking cavernous haemangioma MRI on T1 (left) and T2 (right) sequences. The lesion (yellow arrow) appears hypointense in T1 and hyperintense in T2, interpreted as cavernous haemangioma. MRI: magnetic resonance imaging; HA: hepatic angiosarcoma

Given the atypical radiological findings, a magnetic resonance imaging (MRI) of the liver was deemed necessary to further characterise the lesion. On MRI, the lesion appears hyperintense on delayed images. T2-weighted MRI shows marked hyperintensity, features in keeping with cavernous haemangioma (Figure 4). Despite the patient having had surgery for breast cancer in the past, previous imaging studies were not available to review and confirm the presence of this benign entity within the liver, and the final conclusion was that no further workup or follow-up was required. 

**Figure 5:**
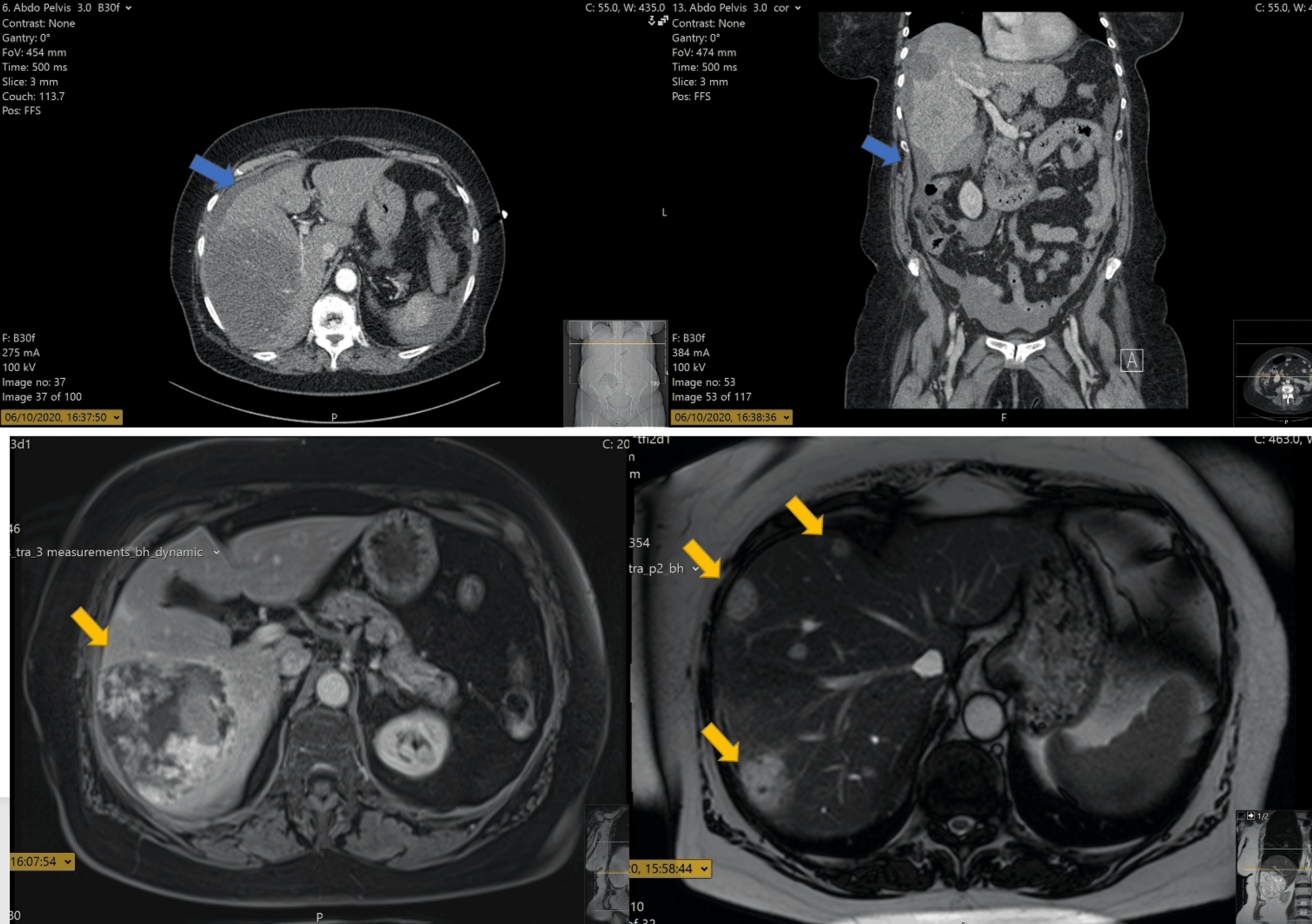
HA mimicking cavernous haemangioma On top, CECT demonstrates spontaneous HA bleeding with perihepatic free fluid evident (blue arrows). On the bottom, MRI imaging shows HA progression in size but also new lesions demonstrating disease spread. MRI: magnetic resonance imaging; HA: hepatic angiosarcoma; CECT: contrast-enhanced computed tomography

One month later, the patient re-presented with intra-abdominal bleeding. CECT revealed rupture of the previously labelled cavernous haemangioma, and treatment involved right hepatic artery embolisation (Figure 5). Furthermore, multiple new liver lesions were identified with guided biopsy, confirming HA. The patient was referred to Sarcoma Oncology services; however, the aggressiveness of the disease did not allow for any additional treatment, and the patient died one month later. 

## Discussion

HA is an aggressive malignancy. Most cases present with advanced disease involving both liver lobes, and distant metastases in lungs and bones may be present [2,12]. Prognosis is poor, with most patients dying within six months of diagnosis [2,12]. Surgical resection and adjuvant systemic treatment have better survival, reaching a five-year survival of 79% in some series [3,12].

Early diagnosis on imaging studies plays a pivotal role, as it provides potential for treatment. In contrast-enhanced CECT, HA typically presents with peripheral enhancement in the late arterial phase and may exhibit a reversed enhancement pattern compared to that of benign cavernous haemangioma [13,14]. While haemangiomas often show early central enhancement followed by delayed peripheral fill-in, the absence of washout usually argues against malignancy. However, imaging features of HA are often inconsistent and may be misinterpreted [13,14].

In the first case described here, retrospective imaging review identified heterogeneous arterial enhancement at the periphery of the cystic lesion, an alarming radiological feature suggestive of aggressive malignant pathology. Nonetheless, these findings were initially overlooked, as the lesion was discovered incidentally during a work-up for persistent cough, and the patient lacked specific hepatic symptoms.

On non-contrast MRI, HA appears hypointense on T1-weighted images and characteristically shows heterogeneous hyperintensity on T2-weighted images [13,15]. This feature can help distinguish HA from benign haemangiomas; hence, careful evaluation and heightened radiological awareness during interpretation are essential [13,15]. In the second case described here, this specific feature was retrospectively identified as the key radiological sign raising suspicion for malignancy. On contrast-enhanced MRI, HA demonstrates imaging characteristics similar to those seen on CECT, including a heterogeneous enhancement pattern in both the arterial and venous phases [12,14,15]. These appearances can resemble those of other vascular liver tumours, and HA may easily mimic a benign haemangioma [15].

As radiological studies fail to contribute to early diagnosis, the rapid progression of disease on repeat imaging studies led to late diagnosis in both cases [12,14]. Similar cases have been reported in the literature, where hepatic angiosarcoma initially mimicked benign lesions such as haemangiomas, cystadenomas, or simple cysts, resulting in diagnostic delay [16,17]. The radiologic overlap between HA and cavernous haemangiomas has also been well reported [15,18]. Bartolotta et al. emphasized the diagnostic pitfalls in distinguishing vascular liver tumours with atypical enhancement patterns, further supporting the need for close imaging follow-up in ambiguous cases [18].

Radiological differentiation between HA and benign hepatic lesions is summarised in Table 1.

**Table 1:** Radiological features of HA and mimics. Radiological features of HA in comparison with common benign hepatic lesions it may mimic, including cavernous haemangioma, simple liver cyst, and biliary cystadenoma. Imaging characteristics are listed across ultrasound, CT, and MRI modalities, with emphasis on enhancement patterns and lesion progression. CT: computed tomography; MRI: magnetic resonance imaging

Feature	Hepatic Angiosarcoma (HA)	Cavernous Hemangioma	Simple Liver Cyst	Biliary Cystadenoma
Echogenicity (US)	Heterogeneous / mixed echogenicity	Homogeneously hyperechoic	Anechoic with posterior enhancement	Anechoic or septated, may have mural nodules
CT (Non-contrast)	Hypodense or mixed attenuation; may contain hemorrhage	Hypodense	Water attenuation (<20 HU)	Cystic lesion, often septated, ± calcifications
CT (Arterial phase)	Irregular peripheral or nodular enhancement, rapid washout	Peripheral nodular enhancement (classic)	No enhancement	Septal or mural enhancement
CT (Portal/venous phase)	Washout of enhancement, progression of necrosis	Progressive centripetal fill-in	No enhancement	Persistent enhancement in septa/nodules
MRI T1-weighted	Heterogeneous, possibly hyperintense (hemorrhage)	Hypointense	Hypointense	Variable: low-to-intermediate signal
MRI T2-weighted	Heterogeneous, high signal with areas of necrosis	Markedly hyperintense	Brightly hyperintense	Hyperintense with septations
Contrast-enhanced MRI	Early irregular enhancement with washout	Peripheral nodular enhancement with fill-in	No enhancement	Septal/mural nodular enhancement
Growth	Rapid progression between studies	Stable over time	Stable	Slow-growing, can increase gradually
Associated findings	Hemorrhage, necrosis, invasion, multifocality	None	None	May have biliary ductal dilation or compression
Clinical correlation	Often symptomatic, rapid deterioration	Incidental, asymptomatic	Incidental	May present with mass effect or vague symptoms

Histological studies may prove useful in the future in identifying HA subtypes potentially curable with liver transplantation, a treatment that is currently not indicated for this aggressive primary liver malignancy [3,9,12]. 

These cases highlight the importance of maintaining a high index of suspicion when encountering hepatic lesions with atypical or ambiguous imaging features. In clinical practice, reliance on a single imaging modality or a one-time scan can delay diagnosis, particularly when initial appearances suggest a benign lesion and it is encountered in patients in their sixth decade of life. Prompt follow-up imaging, especially in the presence of radiological red flags such as heterogeneous enhancement, atypical growth patterns, or interval changes, should be considered even in asymptomatic patients [15,19]. Furthermore, early percutaneous biopsy, although sometimes challenging in vascular tumours, may provide critical diagnostic clarity and should not be deferred in cases with progressive or unexplained imaging findings [20,21]. Adoption of this approach could potentially expedite diagnosis, avoid misclassification, and open a therapeutic window in a malignancy otherwise associated with dismal outcomes [2,22].

## Conclusions

HA is a rare but very aggressive primary liver malignancy. Absence of symptoms shifts the diagnostic burden to imaging studies, with the latter failing to alarm clinicians as the tumour can mimic benign liver pathologies. Clinicians and radiologists should be alert to this pathology in patients in the sixth decade of life when interpreting vague symptoms, if any, and imaging studies with somewhat atypical features. Early diagnosis and surgical resection provide the only chance for prolonged survival.

These cases underscore the importance of prompt follow-up imaging and consideration of early biopsy when radiological features are equivocal or deviate from typical benign patterns. Incorporating such strategies into clinical practice may aid in earlier recognition of this aggressive malignancy, potentially improving outcomes in an otherwise dismal disease. Further histological and molecular analysis of HA subtypes may, in the future, define new therapeutic approaches and identify a subset of patients who may benefit from liver transplantation, a modality currently not recommended in this setting.
